# Effect of Rheological Properties of Polymer Solution on Polymer Flooding Characteristics

**DOI:** 10.3390/polym14245555

**Published:** 2022-12-19

**Authors:** Farhood Navaie, Ehsan Esmaeilnezhad, Hyoung-Jin Choi

**Affiliations:** 1 Department of Petroleum Engineering, Hakim Sabzevari University, Sabzevar 9617976487, Iran; 2 Department of Polymer Science and Engineering, Inha University, Incheon 22212, Republic of Korea; 3 Program of Environmental and Polymer Engineering, Inha University, Incheon 22212, Republic of Korea

**Keywords:** rheology, polymer flooding, thixotropic properties, enhanced oil recovery

## Abstract

Polymer flooding is an appropriate enhanced oil recovery (EOR) process that can increase macroscopic sweep efficiency. We examined two polymeric superpushers at different salinities (10,000 and 42,000 ppm of NaCl and 18,000 ppm of CaCl_2_) and temperatures (30 to 75 °C) as polymer-flooding agents for the EOR process. Rheological and thixotropic tests were attempted to find shear viscosity change when the polymer solutions were introduced under different salinity and temperatures, followed by describing the rheological behavior with the two most common rheological models used for polymer solutions, and then a quadratic model with Design-Expert to detect the effective parameters. Core flooding tests were conducted afterward to determine the final proposed fluid. An increase in the concentration of monovalent ions and the addition of divalent ions adversely affected both types of polymers used, which was similar to the effects of a temperature increase. The Flopaam 3630S at 1000 ppm has more stability under harsh conditions and enables 22% and 38% oil recovery in carbonate and sandstone core rocks, respectively. Consequently, Flopaam 3630S can be the perfect polymer agent for different chemical flooding procedures in high-salinity oil reservoirs.

## 1. Introduction 

Despite the ever-growing call for fossil resources, discoveries and drilling of traditional oil reservoirs are decreasing. Owing to the inefficiency of conventional oil recovery processes, the demand for practical techniques to improve crude oil recovery is essential [1]. This has inspired petroleum researchers to find advanced recovery techniques to increase production from existing reservoirs, where enhanced oil recovery (EOR) techniques are needed [2]. Polymer flooding is a widely used method in the EOR process [3]. It helps oil recovery by changing the macroscopic properties, and thus, optimizing the mobility ratio, which is directly associated with the rheological property of crude oil and a flooding fluid in the reservoir [4]. More precisely, in a polymer flooding operation, the polymer solution, which is considered a viscous fluid in the reservoir medium, pushes the oil to the production wells [5].

A rheological study is essential to observe the intrinsic fluid property of samples by examining the rheological variables of shear stress, shear rate, and shear viscosity from a steady shear test [6]. The resulting flow curve is important for analyzing materials, such as Newtonian and non-Newtonian fluids [7,8]. For a time-dependent non-Newtonian fluid, the shear viscosity changes over time depending on the shear path [9,10].

A polymer with potential use in polymer flooding in EOR needs to be water-soluble with the desired rheological behaviors. Such a polymer needs to increase the volume of displaced fluids and allow good mobility control so that the rheological behaviors of the flooding fluid after being injected into the reservoir will improve the oil-displacing efficiency [11,12]. 

On the other hand, thixotropy, which is an essential part of rheology, is defined as a shear viscosity decrease with time. The shear viscosity then recovers when the applied shear rate is stopped [13]. Most thixotropic fluids are shear thinning [14]; therefore, a thixotropic sample thins with time, requiring decreasing shear stress to keep a constant shear rate. Its physical explanation relies on the particle interaction forces determining the potential energy well for each particle in the case of the colloidal suspension of paints [15]. In addition to considering all the above points, in a polymer flooding process, polymer solution as a flooding fluid with a higher viscosity than water is injected into the oil reservoir to push the oil to the production well. One of the key factors that affect the performance of polymer flooding is the thermal stability of the polymer solution at high temperatures [16]. Therefore, a laboratory study of the thermal stability of the polymer is necessary.

One of the recent works on the high temperature and high salinity polymer flooding test showed that the SAV10, a rich AMPS copolymer recently developed by SNF, can tolerate high salinity brine and temperatures up to 120 °C [16]. It is also demonstrated that different partially hydrolyzed polyacrylamides (HPAMs) can stand oil reservoir conditions (high salinity/high temperature) and enhance the oil recovery up to 30% even in a low polymer concentration [17,18]. Despite these studies, an exhaustive and more detailed experimental study of the scope of HPAM in the EOR is necessary to provide new insight into a practical application of polymers related to EOR.

In this study, two of the most applicable polymers in EOR were selected, and the rheological and thixotropic properties of their solutions in different concentrations were investigated and modeled using two constitutive models in this field. Owing to the conditions of the oil reservoirs, the effects of salinity (concentration and salt type) and different temperatures were investigated to choose the better one to evaluate the feasibility and compare the two common types of polymers for EOR by comprehensive rheological tests. A core flooding test was then performed with the aim of obtaining a better option efficiency in enhancing oil recovery. Furthermore, both resistance factor (RF) and a residual resistance factor (RRF) were evaluated to clarify the consequence of Flopaam 3630S on the EOR.

## 2. Experimental Section

### 2.1. Material

Two kinds of polymers with commercial names, B192 (anionic, low molecular weight, 0.8 kg/m^3^ bulk density) and Flopaam 3630S (anionic, ultra-high molecular weight, 0.67 kg/m^3^ bulk density), were purchased from the SNF Floerger company, France. The salts of both NaCl and CaCl_2_ (Merck Co., Ltd. Darmstadt, Germany), mentioned in the technical data, were reported by the manufacturer. Polymer solutions were prepared by the API RP 63 (Recommended Practices for Evaluation of Polymers Used in EOR processes standard by American Petroleum Institute, 1990) standard approach [19]. The crude oil (API of 32.292° and density of 0.83 g/cc at 15.6 °C) applied to the test beds of carbonate and sandstone was obtained from a crude oil field in a southwestern part of Iran.

### 2.2. Rheological Measurement

The rheological characteristics of the aqueous polymer solutions were investigated via a rotation viscometer (RST-CC Brookfield, Middleborough, MA, USA) at 25 °C. A concentric cylinder modification (CC3-40) was used because of its higher measuring accuracy. This apparatus drives the spindle in the sample holder that contains the test fluid sample and can provide a shear rate [20].

The main drawback of a high polymer concentration relates to the injectivity problem [21], whereas a low polymer concentration cannot increase the macroscopic efficiency. Therefore, three optimal concentrations of 500, 1000, and 1500 ppm were selected for the polymer concentration to investigate the rheological properties, and two more common polymer concentrations (1000 and 1500 ppm) were selected for further experiments [22]. Different fluids (such as freshwater (distilled water), low salinity (10,000 ppm NaCl), high salinity (42,000 ppm NaCl), and simulated Persian Gulf waters (42,000 ppm NaCl and 18,000 ppm CaCl_2_) [23]) were prepared as a solvent for the polymer solution to comprehensively examine the effect of salinity. Here, the following Herschel–Bulkley equation was adopted to fit the flow curve of the polymer solution samples [24,25]

(1) $$\tau =\tau_{y}+K{\dot{\gamma }}^{n}$$

where τ, τ_y_, K, $\dot{\gamma }$, and n are shear stress, yield stress, consistency coefficient, shear rate, and flow index, respectively [26,27]. 

The Bingham fluid equation was also employed [28] as follows:

(2) $$\tau =\tau_{y}+\eta_{o}\dot{\gamma }$$

where η_o_ is the plastic viscosity similar to shear viscosity at a high shear rate [25].

A large domain of temperatures was considered from 30 to 75 °C to cover all possible temperatures of Iranian oil reservoirs, in order to determine their effect [29]. The thixotropic properties were observed and evaluated using the hysteresis method, and the Herschel–Bulkley equation (Equation (1)) was applied to study the thixotropic behavior.

This procedure selected the proposed polymer solution for the flooding core sample. To verify the result with both statistical and experimental methods, the D-optimal and the analysis of variance (ANOVA) methods were used to examine the parameter effects and propose the best sample fluid for the subsequent core flooding tests. In the ANOVA method, diverse regression models may be proposed, depending on the parameters and their interaction. The best model could be obtained by using a residual analysis and dispersion of the response. The D-optimal process as a technique requires model selection at the outset. The most generally considered method in this study was a quadratic model [30]. The D-optimal process can modify the desired model as intended.

Using the finally proposed fluid, the core flooding test was followed to find its capability as an EOR technique.

### 2.3. Preparation and Characteristic of Core Sample

**Figure 1:**
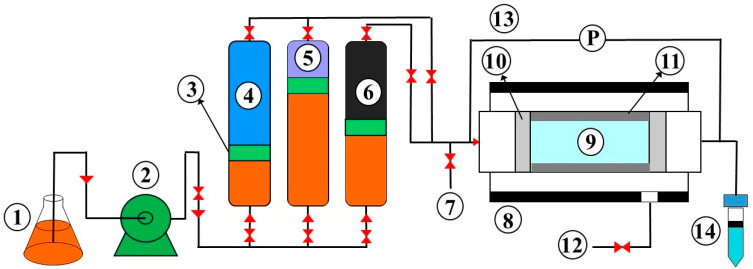
Schematic diagram of core flooding tester: (1) hydraulic oil, (2) pump, (3) piston plate, (4) water or brine accumulator, (5) polymer accumulator, (6) oil accumulator, (7) bypass valve, (8) core holder, (9) core plug, (10) distributor, (11) sleeve, (12) confining pressure, (13) pressure gauge, (14) outlet.

One carbonate core sample and a sandstone core sample were arranged for a regular core flooding test. The core plugs were provided using a core plugging component and a cutter (Fars EOR Co., Shiraz, Iran). Their porosity and gas permeability were achieved (Table 1) via an unsteady-state gas permeation tester (Petro Pazhouhesh Ahoura Co., Shiraz, Iran). The flooding operation was carried out using a common setup of core flooding measurements, of which Figure 1 presents its schematic diagram.

In the subsequent stages, the core plug was wiped clean with deionized water (DW) to extract dirt and placed in the vacuum oven (65 °C) for 8 h until reaching the constant weight that showed the cores were completely dried. The carbonate and sandstone cores were then placed in a synthetic brine solution for one month for spontaneous imbibition. Subsequently, a synthetic brine was inserted into the core in two stages from each of their two ends at a 1 cm^3^/min flow rate (1 ft/day: corresponding to the standard front rate in the field [31]), achieving as much as full saturation regarding the forced imbibition. Darcy’s law (Equation (3)) was adopted for the core brine that was flooded to calculate the liquid permeability [20]:

(3) $$Q=\frac{\mathrm{KA}}{\mu }\frac{\mathrm{dP}}{\mathrm{dx}}$$

where Q, A, K, dP/dx, and *μ* are the flow rate (cm^3^/s), cross-sectional area (cm^2^), permeability (D), and pressure gradient within the core direction (atm/cm), and shear viscosity (mPa.s), respectively. Both cores were aged in the sample crude oil for fourteen days and flooded with crude oil at 0.5 cm^3^/min until no more water was produced, after which they were used to simulate the EOR technique. An artificial brine solution was adopted to perform each water flooding and post-flush water flooding test, whereas the proposed fluid was adopted for flooding with the polymer solution. The results of the oil produced and the differential pressure (ΔP) were obtained with time during the test. Equation (4) [32] was adopted to estimate an oil recovery factor:

(4) $$\mathrm{Cumulative}\;\mathrm{Oil}\;\mathrm{Recovery}\;\left(\%\right)=\frac{\mathrm{Produced}\;\mathrm{oil}\;\left(\mathrm{mL}\right)}{\mathrm{Oil}\;\mathrm{in}\;\mathrm{place}\;\left(\mathrm{mL}\right)}\times 100$$

A two-percent error in the ultimate values was assumed considering the high differential pressures recorded and the relevant oil recovery factor calculation.

## 3. Results and Discussion

### 3.1. Rheological Measurements

**Figure 2:**
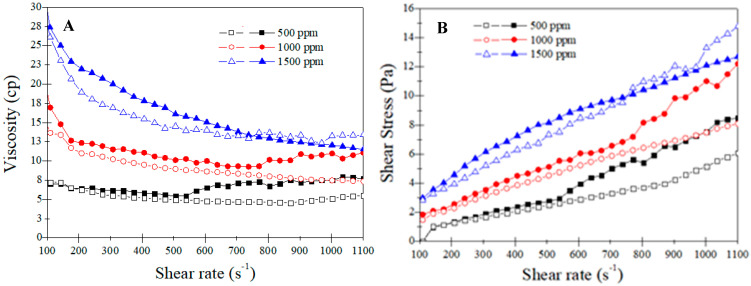
(**A**) Shear viscosity and (**B**) shear stress versus shear rate for B192 (open) and Flopaam 3630S (closed).

A common shear rate sweep test was conducted from 100 to 1100 S^−1^ to investigate the rheological characteristics of the polymer solutions. Figure 2 presents the viscosity and shear stress versus the shear rate for both samples at different concentrations. Both samples showed shear-thinning behavior, especially in higher concentrations. The molecular alignment under shear would provide an easier flow of the polymer chains, decreasing the shear viscosity with an increased shear rate [33,34]. 

Flopaam has a higher viscosity, particularly in a low range of shear rate, which is more dominant in the porous media of the oil reservoir. This is probably due to the higher molecular weight than B192 [35]. Hence, Flopaam is a better choice for polymer flooding and can produce a better mobility ratio in the oil reservoir. An increase in polymer concentration had a great impact on enhancing viscosity, but the effect was more intense between 1000 and 1500 ppm; Flopaam showed a more uniform increase.

The Herschel–Bulkley model showed the best fit for all the prepared polymer solutions (Table 2). In this case, “R^2^” ranged between 0.95 and 0.97 for B192 and from 0.96 to 0.98 for Flopaam 3630S. 

Table 2 lists the fitting parameters of the Herschel–Bulkley equation. The flow index “n” values were correlated with the degree of pseudo-plasticity; smaller values led to significant shear thinning, which was not noticeable in these polymers. The yield stress and consistency index also increased with increasing concentration (Table 2). Thus, for the Flopaam 3630S with the maximum concentration, τ_o_ and K increased approximately sevenfold compared to the minimum concentration, while the above parameters increased less for B192. This behavior of the employed polymers, specifically Flopaam 3630S, is explained by the decreased mobility of the polymeric chains and the increase in the lifetime of the polymeric chain clusters [36]. 

The optimal fitting parameters of the Bingham fluid equation are listed in Table 3. The Bingham model covered just the 1000 and 1500 ppm solutions.

### 3.2. Concentration and Salinity Examination

**Figure 3:**
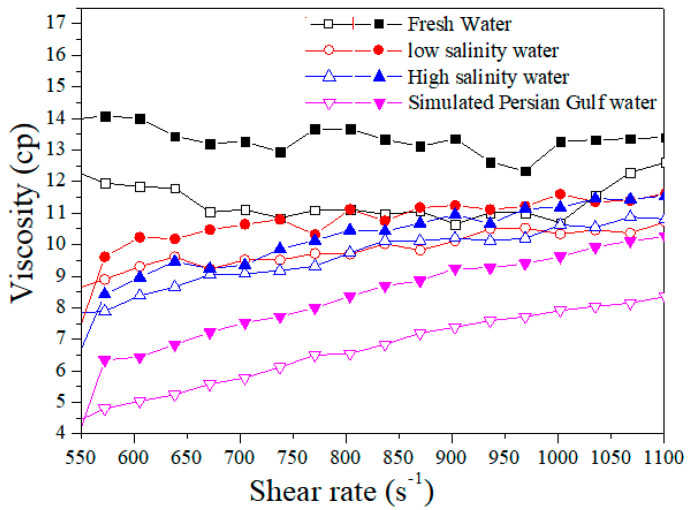
Viscosity vs. shear rate at different salinity for 1500 ppm (closed) and 1000 ppm (open) of Flopaam 3630S.

A common shear rate sweep test concerning concentration and salinity was conducted from 550 to 1100 S^−1^ to investigate the polymer solutions. Initially, the effects of polymer concentration and salinity on the Flopaam 3630S were studied. As is obvious in Figure 3, the viscosity increased with an increasing polymer concentration. By adding salt, the first sharp reduction occurred after adding 10,000 ppm of NaCl. Increasing the salt concentration to 42,000 ppm of NaCl reduced the viscosity further, but at a much slower rate. The second sharp reduction in viscosity occurred after adding a divalent ion (CaCl_2_). Hence, the divalent salt type has a larger effect than the monovalent ion concentration. The divalent ions, considering the higher charge and polarizability, link more firmly to the polyelectrolytes to induce a greater reduction in viscosity than the monovalent ion [37]. Divalent ions can also cause a decrease in the viscosity of the polymer solution because of the bridging effect [38]. The viscosity of the HPAM polymers is particularly dependent on their molecular weight, chain structure, chain conformation, and chain aggregate. Changes in the chain structure and molecular weight may be due to either breaking or combining polymeric chains through covalent bonds in the presence of salt [39]. The addition of salts altered the chain aggregation and conformation through ionic and hydrogen bonds. A similar trend was observed for B192. These results concurred with the literature [5,40].

**Figure 4:**
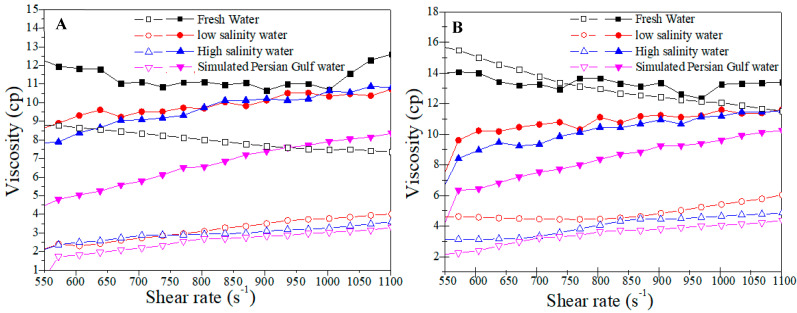
Viscosity vs. shear rate at different salinity for Flopaam 3630S (closed) and B192 (open) polymer solutions at (**A**) 1000 ppm and (**B**) 1500 ppm concentrations.

Two types of polymer in two different concentrations were compared to realize their resistance to salinity, which was conducted at four salinity levels to understand the effects of this parameter effect. Figure 4 shows a reduction in viscosity after adding salt to both polymer solutions. In the case of B192, however, it was worse, and the viscosity was reduced more against Flopaam 3630S. The divalent ion (CaCl_2_) had greater effects on B192 than Flopaam 3630S. Hence, Flopaam 3630S is a better choice in a saline reservoir because it can maintain its rheological properties better than B192 and act as a better polymer agent in a polymer flooding operation. A comparison between the two parts of Figure 4A,B showed that salinity and the polymer concentration are synergic effects because the viscosity reduction is greater in high-concentration cases.

Statistical methods were used to verify the results of the experimental technique, so a D-optimal technique and the analysis of variance (ANOVA) were applied to scrutinize the parameter effects and identify the superior polymer in terms of performance and salinity resistance.

Two factors were assessed: polymer concentration (two levels: 1000 and 1500 ppm) and salinity (four levels: freshwater, low and high salinity, and simulated Persian Gulf water). The levels of each parameter were chosen based on the sensitivity analysis explained above. According to the number of possible solutions (eight), the Design-Expert software underwent eight runs for each polymer. The shear viscosity at a 1000 s^−1^ shear rate is listed for B192 and Flopaam 3630S in Table 4.

The effects of each individual parameter and the changing level of each parameter were examined using an ANOVA for a quadratic model (Table 5). The model *p*-value of <0.0001 for both polymers indicates that the models are significant, considering the R-squared values of 0.9984 and 0.9773 for B192 and Flopaam 3630S, respectively. The predicted R-squared of 0.9870 and 0.9173 for B192 and Flopaam 3630S, respectively, has a logical connection with the adjusted R-squared of 0.9235 and 0.8725, with a difference of less than 0.2. Focusing on the *p*-value < 0.05, which is the global standard of engineering calculation [41], the outstanding performance of the superpushers, especially Flopaam (*p*-values < 0.0001), in advancing the rheological behaviors can be confirmed because salinity has minor effects on viscosity (*p*-values = 0.002 and 0.01 for B192 and Flopaam 3630S, respectively). This phenomenon of the insignificant effect of salinity on shear viscosity can be attributed to the slight molecular chain collapsing [33].

### 3.3. Temperature Effect

**Figure 5:**
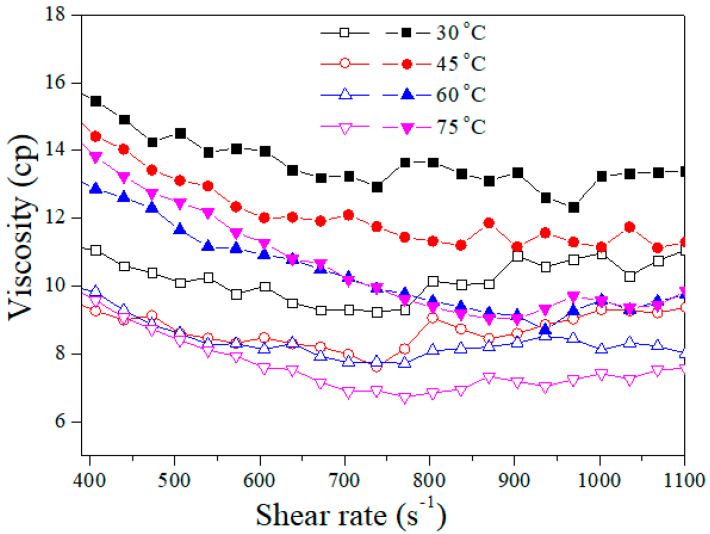
Shear viscosity of Flopaam 3630S solution at 1500 ppm (closed) and 1000 ppm (open) at different temperatures.

**Figure 6:**
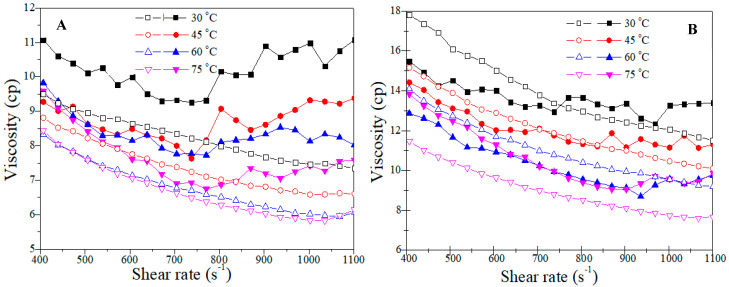
Shear viscosity of Flopaam 3630S (closed) and B192 (open) polymer solutions of (**A**) 1000 ppm and (**B**) 1500 ppm concentration at different temperatures.

After preparing the samples and installing the instruments, a shear rate sweep test was conducted from 400 to 1100 S^−1^ by bob-and-cup geometry in a rheometer to determine the rheological characteristics of the polymer solutions at different temperatures. Initially, the effect of temperature on the Flopaam 3630S polymer solutions was studied at two concentrations. As shown in Figure 5, an increasing polymer concentration (at the same temperature) leads to an increase in shear viscosity that is clear and predictable. After increasing the temperatures to 45 and 60 °C, the viscosity decreased sharply, but at 60 °C, the solution lost its thermal stability, and at higher temperatures (at 75 °C), there was no change. After 60 °C, the polymer agent lost its properties, and the solution behaved like a pure Newtonian fluid with further increases in temperature. A similar trend was observed for B192, as shown in Figure 6. These results followed the literature [16,42]. 

Subsequently, to judge the thermal stability of the two selected polymers, a comparison was carried out at two different concentrations at various temperatures to determine the chosen polymer’s thermal resistivity under oil reservoir conditions.

Based on the flow curve of both types of polymers at two concentrations (Figure 6), the viscosity reduced sharply with increasing temperature. This reduction was sharper for the B192 polymer solutions in both concentrations, and the Flopaam 3630S polymer solutions showed better thermal stability. Another important point is that the threshold of thermal stability for these two types of polymers is different. As shown in Figure 6A, the B192 polymer solution at 1000 ppm lost its thermal stability at 60 °C, and at a higher temperature, it did not show any change in viscosity. This threshold for the Flopaam 3630S polymer solution occurred at 1500 ppm (Figure 6B). It can be deducted from the analysis that using these types of polymers in the oil reservoir, which has a temperature higher than 60 °C, is not useful. Furthermore, Flopaam 3630S has better thermal stability than B192 and is a better candidate for flooding in high-temperature oil reservoirs.

### 3.4. Investigation of Thixotropic Properties

**Figure 7:**
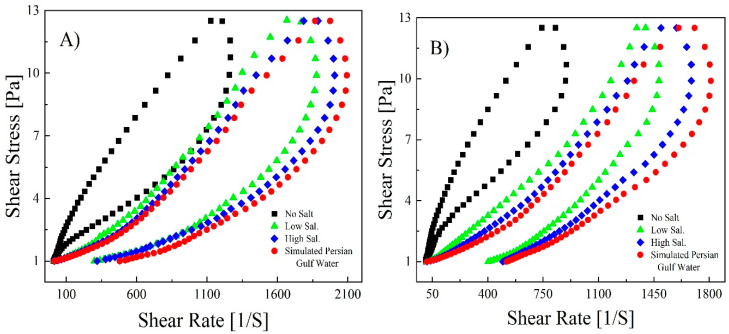
Thixotropic curves of B192 polymer solution at a concentration of 1000 ppm (**A**) and 1500 ppm (**B**) at different salt concentrations.

**Figure 8:**
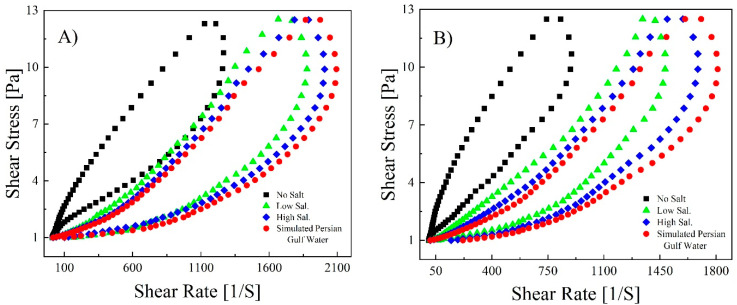
Thixotropic curves of Flopaam 3630S polymer solution at a concentration of 1000 ppm (**A**) and 1500 ppm (**B**) at different salt concentrations.

The fluids (1000 (Figure 7A) and 1500 ppm (Figure 7B) of B192 and 1000 (Figure 8A) and 1500 ppm (Figure 8B) of Flopaam) were prepared, and thixotropic curves were obtained.

The hysteresis curves of both polymers were almost at their proper state and complete position when they covered the path of build-up and breakdown, starting from zero and ending there. The hysteresis curves of Flopaam in Figure 8 show that it passed over its path better than B192 and displayed greater thixotropic properties. Even though it does not provide the intrinsic behavior of any rheological parameter, the hysteresis loop curve is a simple quantitative tool with adequate credibility showing which fluids have thixotropic properties [43]. After completing the rheological tests, the addition of divalent ions to the solutions (high salinity and the simulated Persian Gulf in comparison to low salinity) had a minor effect on the viscosity. Hence, after adding salt, the thixotropic properties of both the 1000 and 1500 ppm polymers decreased the area of the hysteresis loop, which is considered to be the degree of thixotropy [44]. 

The resulting data of B192 were introduced to the Herschel–Bulkley equation and R^2^ > 0.92, indicating that the curves can be fitted well. Table 6 lists the parameter values for B192; similar results are expected for Flopaam. All the samples exhibited positive yield stress for the upper and down curves, indicating that the pristine and branched polymer solutions belong to pseudoplastic fluid with yield stress. Compared to the K values in the upper curve, those in the down curves were greater, showing that the recovery of entanglements was time-independent, and the polymer structure was rebuilt during this test. In the upper curves, the K value increased with increasing polymer concentration, indicating that the pseudo-plasticity of the polymer was strengthened [45].

In addition, a negative value of the hysteresis loop area presented the damage to the polymeric chain structure with an increased shear rate, whereas a positive value represented its structural recovery [46]. The hysteresis loop area of 1500 ppm of the polymer was larger than 1000 ppm, showing that the thixotropic properties and time-dependent behavior decreased with a decreasing polymer concentration. In turn, this showed that the greater the concentration, the greater the binding to the site, so the degree of entanglement of the polymer was high because of this time-dependent behavior of the disentanglement–entanglement process [47] and the alignment of the polymer chain along the shear direction [48].

### 3.5. Core Flooding

According to the rheology experiments and various variables (such as concentrations, salinity, and temperature), and considering the costs of providing the polymer and the small difference between the viscosities in different concentrations according to Figure 4, we determined the optimal concentration of the Flopaam 3630S (1000 ppm) in a high salinity state at ambient temperature for performing the core flooding test in carbonate and sandstone rock cores.

**Figure 9:**
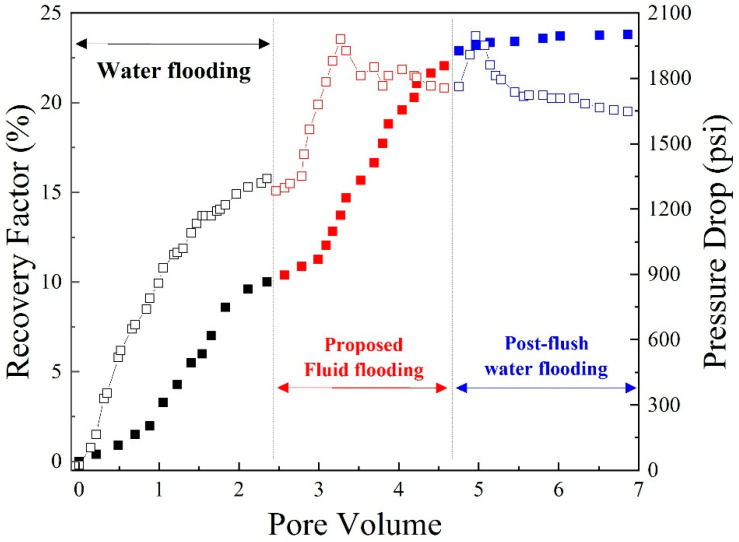
Pressure drop (open) and oil recovery factor (closed) vs. pore volume in Fn-600 core (proposed fluid: 1000 ppm polymer at high salinity).

Figure 9 displays the differential pressure and recovery factor for the Fn-600 core. The EORs after water flooding, polymer flooding (about two-pore volume (PV)), and post-flush water flooding were 10%, 22%, and 22.2%, respectively. It should be mentioned that, up to now, very few polymer flooding projects have used more than 1 PV of the solution, but in this laboratory experiment, because the latter goal is to find RF and RRF, around 2 PV of the polymer solution was injected to study the pressure trend more clearly. At each flood stage, the differential pressure approached a constant threshold associated with the breakthrough phenomenon [20]. The polymer solution raised the macroscopic sweep efficiency; thereby, the EOR improved from 10% to 22%. In the post-flush water flooding stage, the pressure decreased significantly due to the breakthrough or fingering of the polymer solution through the core test bed, providing an easy path through the core. In addition, according to the values of the recovery factor, in both stages of water flooding, no more oil recovery was possible.

**Figure 10:**
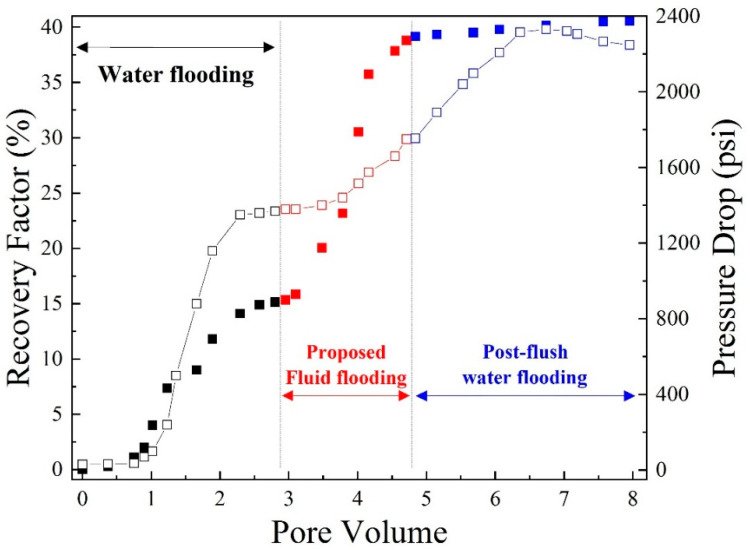
Pressure drop (open) and recovery factor (closed) vs. pore volume in Fn-700 core sample (1000 ppm polymer at high salinity).

As Figure 10 demonstrates, the corresponding results of the EOR parameter for water flooding, polymer flooding (approximately 2 PV), and post-flush water flooding in the Fn-700 core sample were 15.2%, 38.0%, and 40.0%, respectively. Two PV polymer flooding showed a larger EOR factor of 38%. The immutability of the differential pressure at every flooding part indicates the breakthrough phenomenon. In the post-flush water flooding, the pressure drop decreased to a critical value, which is larger than the first stage of water flooding because of a degree of permeability reduction due to polymer adsorption.

These experiments show that Flopaam 3630S improves the EOR by 12% (carbonate core) and 23% (sandstone core) compared to their water flooding tests. On the other hand, the basic mechanism of EOR induced by the added polymer is the promotion of the macroscopic sweep efficiency and enabling a better mobility ratio, where it may alter the wettability and reduce IFT in the sandstone core to reach higher oil recovery [49,50].

Polymer adsorption on a rock surface during flooding is a significant point in polymer flooding, which can affect the oil displacement and reservoir rock properties and subsequent oil recovery [51]. Moreover, polymer retention caused by porous media and polymer chain interactions could induce injectability problems such as additional pressure to inject the polymer solution [18]. This side effect of polymer flooding needs to be considered for its EOR application. Two terms RF and RRF were introduced to evaluate the adsorption in the proposed fluid flooding stage and post-flush water flooding stage, respectively. RF is the ratio of polymer solution flooding differential pressure to the first water flooding differential pressure. Similarly, RRF describes the proportion of differential pressures for post-flush water flooding to primary water flooding [52].

Mobility reduction, formation blockage, or the flow resistance acquired by the proposed fluid consists of increased viscosity and reduced permeability, matching with the term RF. Correspondingly, RRF reveals the permeability reduction of post-flush water flooding [31].

**Figure 11:**
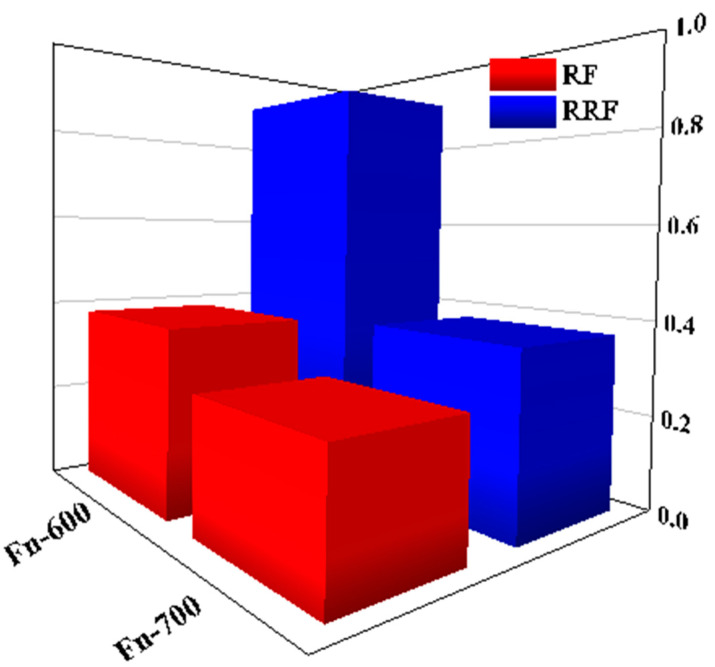
RF and RRF of carbonate core (Fn-600) and sandstone core (Fn-700).

Figure 11 shows the RF and RRF values. The carbonate core (Fn-600) had higher RF and RRF values than the sandstone core (Fn-700). This indicates that the proposed fluid had lower adsorption on the sandstone rock so that better remaining permeability can be expected from the polymer solution, which could be a proper feature to prevent damage formation in the oil field.

## 4. Conclusions

EOR methods are needed to achieve economical and reasonable oil production, where polymer flooding is an efficient and successful method if it can satisfy the screening criteria. This study assessed the feasibility of using two polymers for polymer flooding, and the rheological data were modeled using two common models. However, one of the main drawbacks of the polymer flooding process is its ability to resist salinity and high temperature in the oil reservoir, so these conditions were applied. The results show that the selected polymers can be used in the polymer flooding method to increase the macroscopic efficiency. The rheological behavior could be well estimated using the Bingham and Herschel–Bulkley models. Similarly, the results showed that Flopaam 3630S is a better choice in the case of high saline and high-temperature oil reservoirs than B192 and can work as a flooding fluid in the Iranian oil reservoir in the Persian Gulf. Ultimately, all of the polymer solutions demonstrated specific thixotropy, and various concentrations of polymer and different concentrations and salts caused variations in the thixotropic characteristics. The experiments were followed by flooding the selected fluid in carbonate and sandstone core samples at ambient temperature to analyze the previously found ability in porous media. The proposed fluid increased oil recovery by 22% and 38% in carbonate and sandstone oil reservoirs, respectively. These results will be efficient and beneficial in studies assessing the feasibility of flooding the mentioned commercial polymer for the EOR process. 

## Figures and Tables

**Table 1:** Parameters of the cores used.

Core Name	Core Type	Length (cm)	Diameter (cm)	Porosity (%)	Gas Permeability (mD)	Liquid Permeability (mD)
Fn-600	Carbonate	6.97	3.73	18.65	35.79	8.02
Fn-700	Sandstone	7.08	3.72	8.59	35.31	5.46

**Table 2:** Parameter values of samples using Herschel—Bulkley model.

Polymer	Concentration (ppm)	τ_o_ (Pa)	k	n	Adj. R-Square
Flopaam 3630S	500	0.37	0.00005	1.74	0.98
	1000	2.13	0.00014	1.57	0.97
	1500	2.69	0.00037	1.49	0.96
B192	500	0.68	0.00002	1.75	0.95
	1000	0.70	0.00749	0.99	0.96
	1500	1.10	0.06379	0.75	0.97

**Table 3:** Parameter values of samples using Bingham model.

Polymer	Concentration (ppm)	τ_y_ (Pa)	η_o_ (Pa.S)	Adj. R-Square
Flopaam 3630S	1000	0.09	0.0109	0.94
	1500	2.53	0.0099	0.97
B192	1000	0.071	0.0072	0.96
	1500	2.53	0.0099	0.96

**Table 4:** Experimental design value with corresponding response (shear viscosity) for B192 and Flopaam 3630S.

B192 Run	Factors		Viscosity (cp)
	Polymer Conc. (ppm)	Salinity	
1	1000	No Salt	7.46
2	1000	Low Sal	3.76
3	1000	High Sal	3.25
4	1000	Persian Gulf	3.02
5	1500	No Salt	12.05
6	1500	Low Sal	5.42
7	1500	High Sal	4.64
8	1500	Persian Gulf	4.04
**Flopaam Run**	**Factors**		**Viscosity (cp)**
	**Polymer Conc. (ppm)**	**Salinity**	
1	1000	No Salt	10.69
2	1000	Low Sal	10.62
3	1000	High Sal	10.03
4	1000	Persian Gulf	7.91
5	1500	No Salt	13.25
6	1500	Low Sal	11.59
7	1500	High Sal	11.17
8	1500	Persian Gulf	9.61

**Table 5:** Results of ANOVA for shear viscosity of B192 and Flopaam 3630S.

B192 Source	Sum of Squares	df	Mean Square	F Value	*p*-Value Prob > F
Model	0.065	4	0.016	37.91	< 0.0001
A-Polymer Conc.	0.020	1	0.020	46.73	< 0.0001
B- Salinity	0.045	3	0.015	34.97	0.002
Residual	4.731E-0.03	11	4.301E		
Cor Total	0.070	15			
**Flopaam 3630 Source**	**Sum of Squares**	**df**	**Mean Square**	**F Value**	***p*-value Prob > F**
Model	24.45	4	6.11	38.80	< 0.0001
A-Polymer Conc.	9.19	1	9.19	59.81	< 0.0001
B- Salinity	17.94	3	5.98	38.93	0.01
Residual	1.54	10	15		
Cor Total	25.98	14			

**Table 6:** Parameters of Herschel–Bulkley model for B192 polymer solution.

Sample	Salinity	Up Curve			
		τ_o_ (Pa)	K	n	R^2^
1000 ppm	No Salt	0.046	0.071	0.727	1.000
	Low Sal.	0.531	1.128	1.558	0.999
	High Sal.	0.472	1.030	1.557	0.998
	Persian Gulf	0.445	1.023	1.549	0.998
1500 ppm	No Salt	0	0.154	0.658	0.999
	Low Sal.	0.700	2.962	1.455	0.995
	High Sal.	0.650	7.441	1.633	0.999
	Persian Gulf	0.566	4.776	1.682	0.999
**Sample**	**Salinity**	**Down Curve**			
		**τ_o_ (Pa)**	**K**	**n**	**R^2^**
1000 ppm	No Salt	1.101	1.162	1.892	0.949
	Low Sal.	0.602	3.890	3.166	0.928
	High Sal.	0.637	2.076	3.219	0.923
	Persian Gulf	0.657	5.024	3.387	0.924
1500 ppm	No Salt	0.962	0.005	1.080	0.947
	Low Sal.	0.306	2.651	2.698	0.946
	High Sal.	0.410	1.107	2.765	0.941
	Persian Gulf	0.490	3.313	2.897	0.930
